# How Does Lower Limb Respond to Unexpected Balance Perturbations? New Insights from Synchronized Human Kinetics, Kinematics, Muscle Electromyography (EMG) and Mechanomyography (MMG) Data

**DOI:** 10.3390/bios12060430

**Published:** 2022-06-18

**Authors:** Ringo Tang-Long Zhu, Pei-Zhao Lyu, Shuai Li, Cheuk Ying Tong, Yan To Ling, Christina Zong-Hao Ma

**Affiliations:** 1 Department of Biomedical Engineering, The Hong Kong Polytechnic University, Hong Kong SAR, China; ringo-tanglong.zhu@connect.polyu.hk (R.T.-L.Z.); peizhao.lyu@connect.polyu.hk (P.-Z.L.); sshuai.li@connect.polyu.hk (S.L.); cheuk-ying.tong@connect.polyu.hk (C.Y.T.); jane.yt.ling@connect.polyu.hk (Y.T.L.); 2 Research Institute for Smart Ageing, The Hong Kong Polytechnic University, Hong Kong SAR, China

**Keywords:** balance perturbation, balance control, onset latency, time to peak, electromyography (EMG), mechanomyography (MMG), skeletal muscle, reactive balance response, compensatory postural adjustment (CPA), waist-pulling perturbation

## Abstract

Making rapid and proper compensatory postural adjustments is vital to prevent falls and fall-related injuries. This study aimed to investigate how, especially how rapidly, the multiple lower-limb muscles and joints would respond to the unexpected standing balance perturbations. Unexpected waist-pull perturbations with small, medium and large magnitudes were delivered to twelve healthy young adults from the anterior, posterior, medial and lateral directions. Electromyographical (EMG) and mechanomyographical (MMG) responses of eight dominant-leg muscles (i.e., hip abductor/adductors, hip flexor/extensor, knee flexor/extensor, and ankle dorsiflexor/plantarflexors) together with the lower-limb joint angle, moment, and power data were recorded. The onset latencies, time to peak, peak values, and/or rate of change of these signals were analyzed. Statistical analysis revealed that: (1) agonist muscles resisting the delivered perturbation had faster activation than the antagonist muscles; (2) ankle muscles showed the largest rate of activation among eight muscles following both anteroposterior and mediolateral perturbations; (3) lower-limb joint moments that complied with the perturbation had faster increase; and (4) larger perturbation magnitude tended to evoke a faster response in muscle activities, but not necessarily in joint kinetics/kinematics. These findings provided insights regarding the underlying mechanism and lower-limb muscle activities to maintain reactive standing balance in healthy young adults.

## 1. Introduction

Falls and fall-related injuries adversely affect about one-third of the older population globally [1]. To avoid a fall, it is vital to make prompt and proper postural adjustments to maintain or recover balance, i.e., keeping the center of body mass (CoM) within the base of support (BoS) [2]. Reactive balance response, or compensatory postural adjustment (CPA), describes how human beings react to a sudden perturbation. It refers to the postural control and the activation of muscles after the central nervous system detects the balance perturbation [3]. Throughout the pathway of motor output, an in-depth investigation of how the multiple lower-limb muscles and joints react rapidly to maintain standing balance is needed, which can facilitate our better understanding of the mechanisms underlying CPAs and the fall-prevention strategies.

CPAs can be rarely assessed in the subjective balance scales or questionnaires. Most of the clinical tests, e.g., the Berg Balance Scale (BBS), the Performance-Oriented Mobility Assessment (POMA), and the Short Physical Performance Battery (SPPB), evaluate only the anticipatory postural adjustments (APAs) by instructing clients to accomplish some predictable balance challenging tasks [3]. An exception is the Mini Balance Evaluation Systems Test (Mini-BEST), which includes the CPA assessment items by suddenly putting the clients’ bodies in anterior, posterior, and lateral inclined postures [4]. The CPAs have been more widely studied in a variety of laboratory equilibrium-disturbing or fall-simulation experiments, where the unexpected perturbations were exerted on different body parts (e.g., shoulder [5], waist/pelvis [6,7,8], foot [9,10]) to disturb the original postural stability in either static (e.g., perturbed quiet standing [6,8,9,10], suddenly tether-released inclined standing [11]) or dynamic (e.g., induced slipping [12] or tripping [13,14] during walking) states. These approaches make it possible to elicit the CPAs and evaluate the reactive balance capability in human beings.

From externally to internally, the whole-body postural sways, the kinematics (e.g., angles) and kinetics (e.g., moments and power) of lower-limb joints, the contraction and activation patterns of lower-limb muscles can all affect how fast the CPAs are made. To quantitatively depict such rapid response, some parameters like the onset latency, the time to peak amplitude, and the rate of change were proposed.

Regarding the whole-body postural sways, previous studies found that the center of pressure (CoP) had larger displacement than the CoM when responding to the unexpected balance perturbation [5,6,15]. In this way, the CoM was kept within the BoS, and the standing balance could be maintained. In addition, the time to peak CoM displacement has been reported to vary following the different directions of unexpected platform movements [10]. Regarding the lower-limb joint angles, the onset latencies of hip, knee, and ankle joint motions were studied: (1) during standing, with perturbation induced by a forward-moving [16] or backward-moving platform [9] in the sagittal plane, and (2) during walking, with perturbation induced by waist-pulling [7]. Regarding the lower-limb joint kinetics, previous studies analyzed: (1) the joint moment responses in the sagittal plane following balance perturbations induced by a backward-moving platform [9], (2) the hip and ankle moment responses [6], and the hip power response [8] in frontal plane following waist-pull perturbations. Pijnappels, et al. [14] also reported a smaller rate of ankle plantarflexion, knee flexion, and hip extension moment development in the sagittal plane in the stance leg of participants who fell after the experimentally induced tripping. However, previous kinetic analyses have put limited focus on the temporal parameters. It remained unclear how fast the multiple lower-limb joint moments and power would react to unexpected standing perturbations. It is expected that we could have a better understanding of how the hip, knee and ankle joints coordinate to maintain standing balance following perturbations, upon studying the exact time when various lower-limb joints begin to react and reach peaks. Further studies are needed.

Regarding the lower-limb muscle electrical activities, previous studies have investigated the muscle’s EMG onset latencies [9,16,17,18,19] and the time to peak of EMG amplitude [17,18,19], following unexpected standing balance perturbations induced by a moving platform. Pijnappels, et al. [13] found that compared to young people, older people showed increased onset latency and decreased rate of EMG rise in the dorsal muscles of the stance leg after unexpected tripping during walking. Previous studies also reported the age-related reduction in the hip abductors/adductors’ rate of EMG rise following unexpected standing balance perturbation induced by the mediolateral waist-pulling [8]. However, most of these studies have only investigated the ankle/knee muscles’ EMG signals; and very limited previous studies have concurrently evaluated the rapid responses of hip abductor/adductors, hip flexor/extensor, knee flexor/extensor, and ankle dorsiflexor/plantarflexor. It is expected that studying multiple lower-limb muscles’ reactions and activation patterns could help further uncover the underlying mechanism of CPAs. In addition to EMG, mechanomyography (MMG) is another technology that can measure the lateral vibration and mechanical activities of skeletal muscles [20]. The onset latencies of EMG, MMG and joint moment signals may enable a more detailed characterization of the motor output pathway, and provide insights on whether the slower balance response is more attributed to the delayed neuromuscular activation, the delayed onset of muscle contraction, or the slower force propagation from muscle to tendon [21]. Thus, MMG may serve as an additional tool to characterize the rapid responses of muscle contractile properties, and merits further studies.

Humans react differently to the varying magnitudes of unexpected balance perturbations. With the increasing perturbation magnitudes, larger lower-limb joint responses and larger amplitudes of muscle activities would be evoked to maintain standing balance [9]. The larger perturbation magnitude could even alter the EMG onset sequence of lower-limb muscles from distal-to-proximal to proximal-to-distal activation [22], and change the pattern of postural adjustment from the “ankle strategy” to the “hip strategy”, “mixed ankle and hip strategy” or “stepping strategy” [23,24]. However, previous studies have mostly reported the effects of different balance perturbations on the choice of responding strategies. It is still unclear whether the faster lower-limb responses are required to resolve a larger balance perturbation, which warrants further investigation.

To fill the above-mentioned research gaps, this study aimed to comprehensively investigate and uncover the more in-depth underlying mechanisms of maintaining standing balance, by investigating how the multiple lower-limb muscles and joints react rapidly following balance perturbations. It would answer the research questions of: (1) how do the onset latencies and the time to peak of the hip, knee, and ankle joints’ kinetic and kinematic data respond to the different magnitudes of waist-pull perturbation in sagittal and frontal planes; and (2) how do the onset latencies, the time to peak and the rate of rise of eight lower-limb muscles’ EMG and MMG data respond to the different magnitudes of waist-pull perturbation in sagittal and frontal planes. It was hypothesized that both the temporal parameters and the rate of change would be different across the eight lower-limb motions (hip abduction/adduction, hip flexion/extension, knee flexion/extension, and ankle dorsiflexion/plantarflexion), across the eight lower-limb muscles (hip abductor/adductor, hip flexor/extensor, knee flexor/extensor, and ankle dorsiflexor/plantarflexor), and across the three different perturbation magnitudes (small, medium, and large).

## 2. Materials and Methods

### 2.1. Participants

A total of 12 healthy young adults aged 18–25 years old (6 males and 6 females) were recruited in this study through convenience sampling. Participants having any neuromuscular, orthopedic, or heart disease were excluded. Ethics approval was granted by the university’s Institutional Review Board (reference number: HSEARS20210122001). All participants signed the written informed consent before experiment. The whole experiment was accomplished in the Human Locomotion Laboratory (Department of Biomedical Engineering, The Hong Kong Polytechnic University). 

### 2.2. Equipment

**Figure 1:**
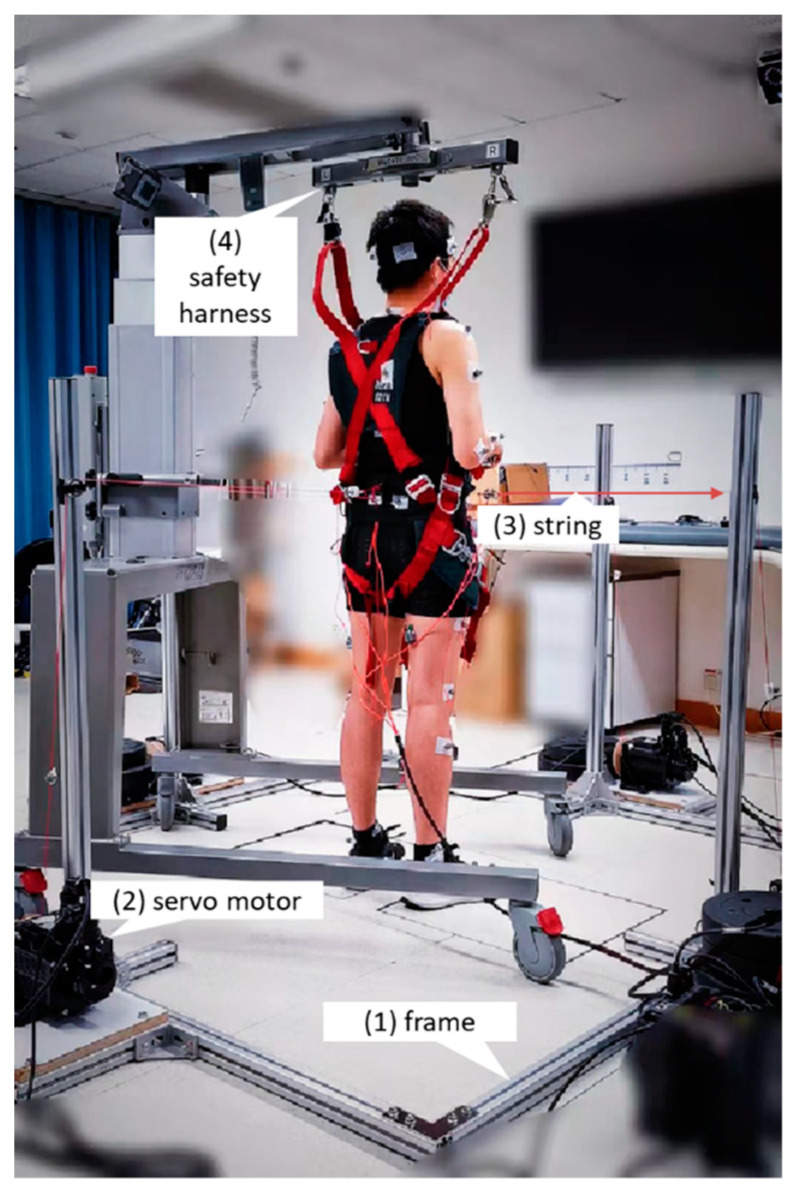
Experimental setup and the waist-pull system for inducing sudden balance perturbations in anterior, posterior, medial, and lateral directions.

As shown in Figure 1, the waist-pull system for inducing balance perturbations mainly involved: (1) an aluminum alloy frame, (2) four servo motors (130-07725AS4, Wenzhou Guomai Electronics Ltd., Wenzhou, China), (3) four pulling strings, and (4) a safety harness. The four servo motors had a rated output power of 2000 W and a rated torque of 7.7 Nm. One end of the pulling string (1.2 mm-diameter braided polyethylene wire) was wired around a 40 mm-diameter driving wheel connecting to the servo motor, and the other end went through a turn on the frame and was tied to the belt worn by the participant at pelvis level. A commercially available harness system (PG-360, Physio Gait Dynamic Unweighting System, Healthcare International Ltd., Langley, WA, USA) was used to prevent the participant from falling during the experiment.

The 3D motion capture and analysis system (Nexus 2.11, Vicon Motion Systems Ltd., Yarnton, UK) was used to collect the pelvic and lower-limb kinematics and kinetics during the experiment. The sampling rates of the eight cameras (Vicon Vantage 5, Vicon Motion Systems Ltd., Yarnton, UK) and the two floor-mounted force plates (OR6, Advanced Mechanical Technology, Inc., Watertown, MA, USA) were 250 Hz and 1000 Hz, respectively. Based on the Plug-in Gait Full-body Model, a total of 39 reflective markers were attached to each participant’s anatomical landmarks, including four on head (bilateral front head, bilateral back head), five on torso (spinous process of the 7th cervical vertebra, spinous process of 10th thoracic vertebra, right scapula, sternal notch, xiphoid process of the sternum), four on pelvis (bilateral anterior superior iliac spine, bilateral posterior superior iliac spine), fourteen on each of bilateral upper limbs (bilateral acromion, upper arm, elbow, forearm, radial side of wrist, ulnar side of wrist, 3rd metacarpal head), and twelve on each of bilateral lower limbs (bilateral thigh, knee, shank, lateral malleolus, heel, 2nd metatarsal head) [25]. The waist-pull system and the Vicon system were synchronized. 

The synchronized eight-channel Trigno Wireless Biofeedback System (SP-W02D-1110, Delsys Inc., Natick, MA, USA) was used for EMG and MMG data collection. Each Trigno Avanti Sensor (dimension: 37 mm × 27 mm × 13 mm; mass: 14 g) comprised an EMG sensor (double-differential silver bar electrodes; electrode size: 5 mm × 1 mm; inter-electrode distance: 10 mm; common mode rejection ratio >80 dB; amplifier gain: 909; analog Butterworth filter bandwidth: 20–450 Hz) and a 9-axis inertial measurement unit which involved a 3-axis accelerometer to serve as the MMG sensor. Based on the Surface ElectroMyoGraphy for the Non-Invasive Assessment of Muscles (SENIAM) guideline [26], eight EMG and MMG sensors were placed longitudinally over the eight lower-limb muscles (Table 1) after skin preparation. The EMG and MMG signals were sampled at 2000 Hz and 250 Hz, respectively.

### 2.3. Protocols

#### 2.3.1. Demographic Data Collection

Each participant’s demographic data, including age, height, and body weight, was collected. Physical activity in the past 7 days and the degree of concerns over falling were evaluated via the International Physical Activity Questionnaire-Short version (IPAQ-S) [29] and the Fall Efficacy Scale-International (FES-I) short version [30], respectively. The participant’s dominant leg was determined as the leg that made a step more often, in response to the six forward and backward shoulder nudges/pushes performed by the researcher [31].

#### 2.3.2. Instrumented Data Collection

During preparation, the waist-pull system and hardness system were set up on each participant, ensuring that: (1) each participant stood with the two feet in shoes and shoulder-width apart on the two force plates separately, (2) the belt was tied just above the height of posterior superior iliac spine (PSIS), and (3) the harness jacket would not restrict anteroposterior or mediolateral postural responses within a certain range [32]. Each participant’s foot positions were then marked with dark-colored tapes, and the length/height of the harness system was fixed. Then, each participant was given five minutes to sit down and rest to avoid fatigue in the following formal perturbed standing trials. 

Before the balance perturbation, each participant was required to hold a light rod in front of the body at the waist level to make sure the reflective markers were detectable. They were instructed to “stand still and look forward; when perturbed by the pulling, try best to maintain postural balance without making steps; if the foot moves, try to return to the initial/original place marked by the dark-colored tapes as soon as possible.” Each participant was also instructed that after the start of pulling, their hands could respond freely [32].

Each participant accomplished three perturbed standing trials with a total of 36 waist-pulls (3 magnitudes × 4 directions × 3 repetitions). The magnitudes (small, medium, and large), the directions (anterior, posterior, medial, and lateral), and the interval time between two pulls (12–15 s) were pseudo-randomized for each participant. Participants were also blinded to the sequence of the waist-pulls during the experiment. Based on the results from our pilot study and the published literature, the maximal anterior, posterior, medial, and lateral pulling displacements were set as the 6% [33], 4% [34], 8%, and 8% [35] of each participant’s height, respectively. The small, medium, and large pulling magnitude corresponded to the 1/3, 2/3, and 3/3 of the maximal pulling displacement, respectively. Each pull’s duration, displacement, and velocity were measured based on the flash time of infrared light, and the movement of the reflective markers fixed on the strings. Videos were taken to record each participant’s behavioral performance during the experiment.

### 2.4. Data Processing

The kinematic and kinetic data (i.e., joint angles, joint moments, joint power, CoM and CoP) were processed by using the Plug-in Gait Dynamic model of the Vicon system [36]. The CoP and joint moments were further filtered using a low-pass 4th order Butterworth filter with a 15 Hz cut-off frequency [37]. The CoM, CoP, joint angle, and joint moment data was zeroed to the mean of the 1000-ms baseline value before each separate pull. 

The muscle activity data as measured by the EMG and MMG sensors was processed by the MATLAB program (MATLAB 2019b, The MathWorks, Inc., Natick, MA, USA). The EMG data were zeroed to the mean values obtained from the whole perturbed standing trial, full-wave rectified, and low-pass filtered using a 4th order and bi-directional Butterworth filter with a cut-off frequency of 4 Hz [38]. To extract MMG data, the accelerometry signals perpendicular to the skin, i.e., the *z*-axis components, were used and processed. The signals were firstly band-pass filtered using a 4th order Butterworth filter (5–50 Hz), then full-wave rectified, and further smoothed via a moving-average filter of temporal window of 0.1 s [39]. The EMG or MMG signal envelope was then normalized to the 1000-ms baseline mean value at the beginning of each perturbed standing trial.

**Figure 2:**
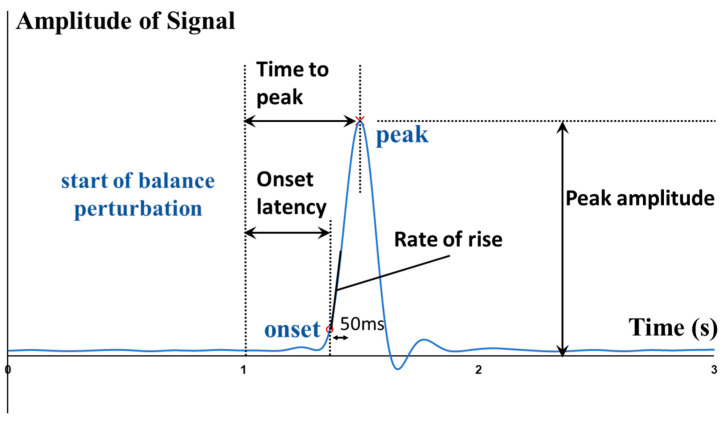
Illustration of the definitions of outcome measures.

The start of balance perturbation was defined as the time point when the motorized waist-pull system started running. The onset and peak points of various signals were identified within 2 s after the start of balance perturbation. The onset time of body CoM, body CoP, joint angle, joint moment, joint power, muscle EMG, and muscle MMG data was defined as the first time point when the corresponding normalized signal/data value went beyond five times of the standard deviation (SD) from the baseline value (mean + 5SD) [40]. The baseline value was calculated as the mean over the 1000-ms interval before the start of balance perturbation. As shown in Figure 2, the onset latencies were referred to as the time delays between the start of balance perturbation and the onset of the corresponding signals. The time to peak was referred to as the delayed time between the start of balance perturbation and the peak of the corresponding signal. The rate of EMG rise was referred to as the slope of EMG signal rise within the 50-ms interval after its onset. 

For each outcome (i.e., onset latency, time to peak, peak value, or rate of change), the three values following the three repeated perturbations in same direction and magnitude were calculated and averaged for each participant. These mean values of the 12 participants were used for the following statistical analysis.

### 2.5. Statistical Analyses

Statistical analyses were performed using IBM SPSS version 25. Intraclass correlation coefficients (ICCs) were calculated to examine the test-retest reliability of three pulls with the same direction and magnitude. Two-way factorial analysis of variance (ANOVA) and post hoc pairwise comparisons with Bonferroni corrections were used to separately examine: (1) the effects of three different perturbation magnitudes and the spatial difference of eight muscles’ electrical and mechanical activities (for EMG and MMG signals; “magnitude” × “muscle”); (2) the effects of three different perturbation magnitudes and the lower-limb joint difference (for angle, moment and power data; “magnitude” × “joint motion”); and (3) the effects of three different perturbation magnitudes and the CoM-CoP difference (“magnitude” × “postural sway”); on the measured onset latency, the time to peak, the specific peak values, and/or the rate of increase. The significance level was set as 0.05. 

## 3. Results

A total of 12 healthy young adults (age: 20.9 ± 0.7 years; gender: 6 males and 6 females; height: 169.9 ± 6.9 cm; weight: 58.3 ± 6.2 kg) participated in this study (Table 2). No fall or other adverse events occurred during experiments, and participants all reported that the harness system did not restrict their movements. As shown in Table 3, the ICC values demonstrated good test-retest reliability of the pulling duration, displacement, and velocity of the waist-pull system in this study. The mean and standard error values across the 12 participants are presented in the figures to illustrate the signal changes (i.e., CoM, CoP, angle, moment, power, EMG or MMG) following perturbations. The range and the standard values across the 12 participants are presented in the Table S1 of Supplementary Materials.

Under the small perturbations, all participants were observed to be able to keep their feet in place. Under the medium perturbations, the stepping of the dominant leg occurred once following the posterior pulls (1 out of totally 36 pulls; 1/36), the elevation of the dominant leg occurred following the medial pulls (1/36), and the elevation of nondominant leg occurred following the lateral pulls (1/36). Under the large perturbations, the stepping or elevation of the dominant leg occurred in one participant following the anterior pulls (3/36), in two participants following the posterior pulls (2/36), in seven participants following the medial pulls (15/36), and in two participants following lateral pulls (3/36); the stepping or elevation of nondominant leg occurred in one participant following the posterior pulls (1/36), in three participants following the medial pulls (8/36), and in five participants following lateral pulls (10/36).

### 3.1. Whole-Body CoM and CoP Displacement

**Figure 3:**
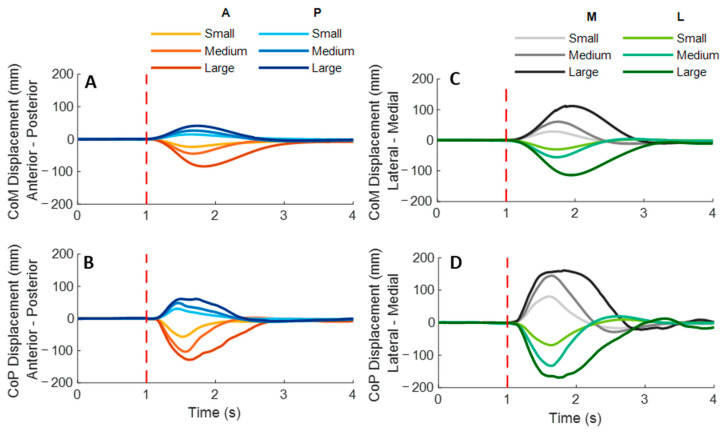
The mean whole-body CoM and CoP displacements of twelve participants following the unexpected anterior, posterior, medial, and lateral perturbations with three magnitudes (*n* = 12). Mean CoM (**A**) and CoP (**B**) displacements following anterior and posterior perturbations; Mean CoM (**C**) and CoP (**D**) displacements following medial and lateral perturbations. (Note: The red dotted line indicated the start of pulling perturbation. **CoM**: center of mass; **CoP**: center of pressure. **A**: anterior; **P**: posterior; **M**: medial; **L**: lateral).

**Figure 4:**
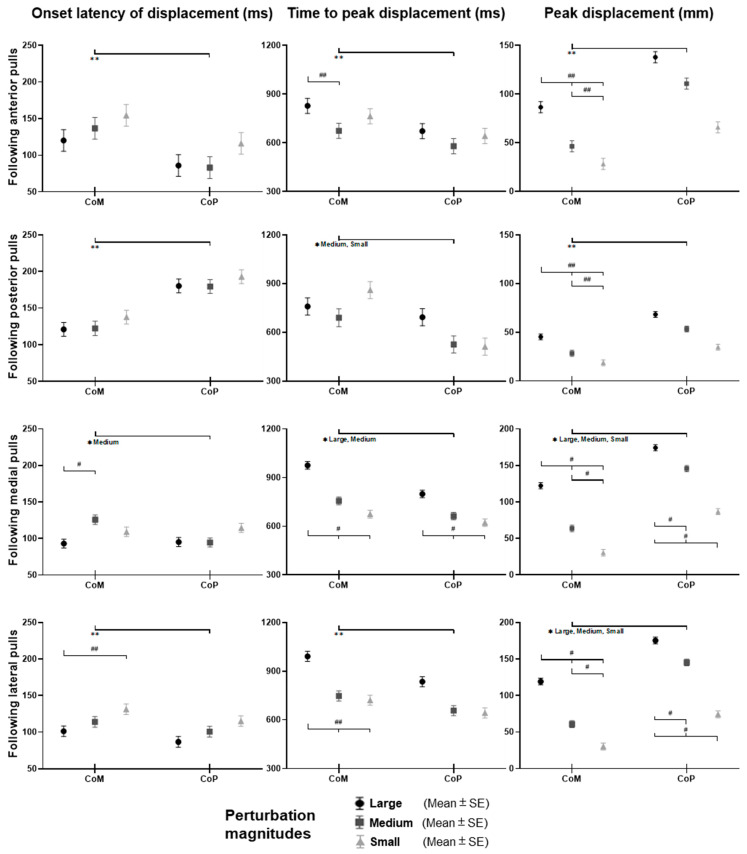
The onset latency of displacement, time to peak displacement, and peak displacement of whole-body CoM and CoP following unexpected horizontal perturbations (mean ± SE, *n* = 12). (Note: **CoM**: center of mass; **CoP**: center of pressure; **SE**: standard error; 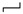 or 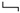: pairwise comparison. Significant differences in post hoc pairwise comparisons (*p* < 0.05) were indicated by the: ** for the main effect of postural sway factor; * for the simple main effect of postural sway factor; **##** for the main effect of magnitude factor; **#** for the simple main effect of magnitude factor).

As shown in Figure 3, the whole-body CoM and CoP displacements mainly moved toward the direction of waist-pull perturbation. As shown in Figure 4, following the unexpected anterior perturbations, CoP showed significantly shorter onset latency of displacement, shorter time to peak displacement, and larger peak displacement than CoM (*p* < 0.05). The larger perturbation magnitudes evoked significantly longer time to peak displacement and larger peak displacements (*p* < 0.05). 

Following the unexpected posterior perturbations, CoP showed significantly shorter onset latency, shorter time to peak displacement under the medium and the small magnitudes, and larger peak displacement than CoM (*p* < 0.05). The larger perturbation magnitudes evoked significantly larger peak displacements (*p* < 0.05). 

Following the unexpected medial perturbations, CoP showed significantly shorter onset latency of displacement under the medium magnitude, shorter time to peak displacement under the large and the medium magnitudes, and larger peak displacement than CoM (*p* < 0.05). The larger perturbation magnitudes evoked significantly shorter onset latency of CoM displacement, longer time to peak displacement, and larger peak displacements (*p* < 0.05).

Following the unexpected lateral perturbations, CoP showed significantly shorter onset latency, shorter time to peak displacement, and larger peak displacement than CoM (*p* < 0.05). The larger perturbation magnitudes evoked significantly shorter onset latency of displacement, longer time to peak displacement, and larger peak displacements (*p* < 0.05).

### 3.2. Lower-Limb Joint Angles and Joint Power

**Figure 5:**
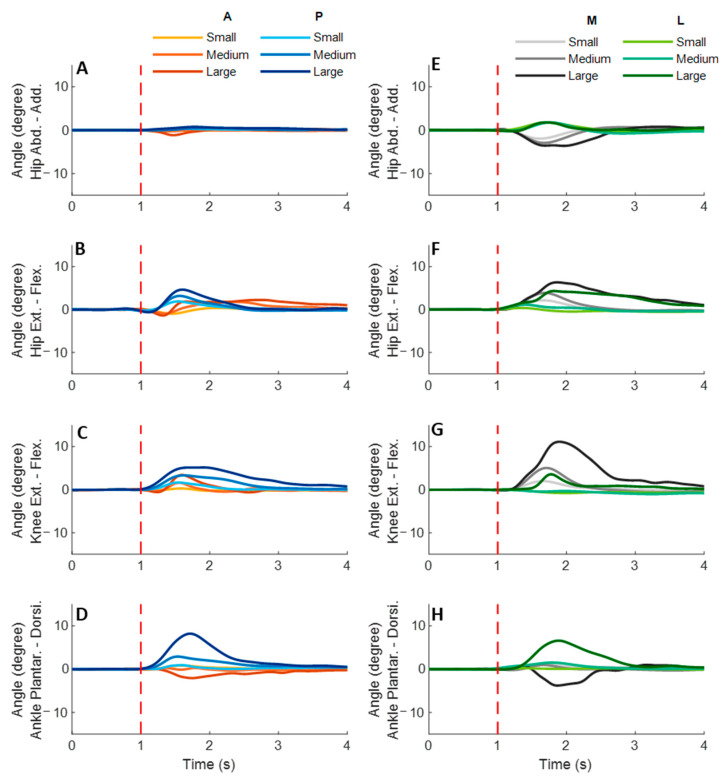
The mean dominant-leg joint angle changes of twelve participants following the unexpected anterior, posterior, medial, and lateral perturbations with three magnitudes (*n* = 12). Mean hip adduction-abduction (**A**), hip flexion-extension (**B**), knee flexion-extension (**C**), and ankle dorsiflexion-plantarflexion (**D**) angle changes following anterior and posterior perturbations; Mean hip adduction-abduction (**E**), hip flexion-extension (**F**), knee flexion-extension (**G**), and ankle dorsiflexion-plantarflexion (**H**) angle changes following medial and lateral perturbations. (Note: The red dotted line indicated the start of pulling perturbation. **Add.**: adduction; **Abd.**: abduction; **Flex.**: flexion; **Ext.**: extension; **Dorsi.**: dorsiflexion; **Plantar.**: plantarflexion. **A**: anterior; **P**: posterior; **M**: medial; **L**: lateral).

**Figure 6:**
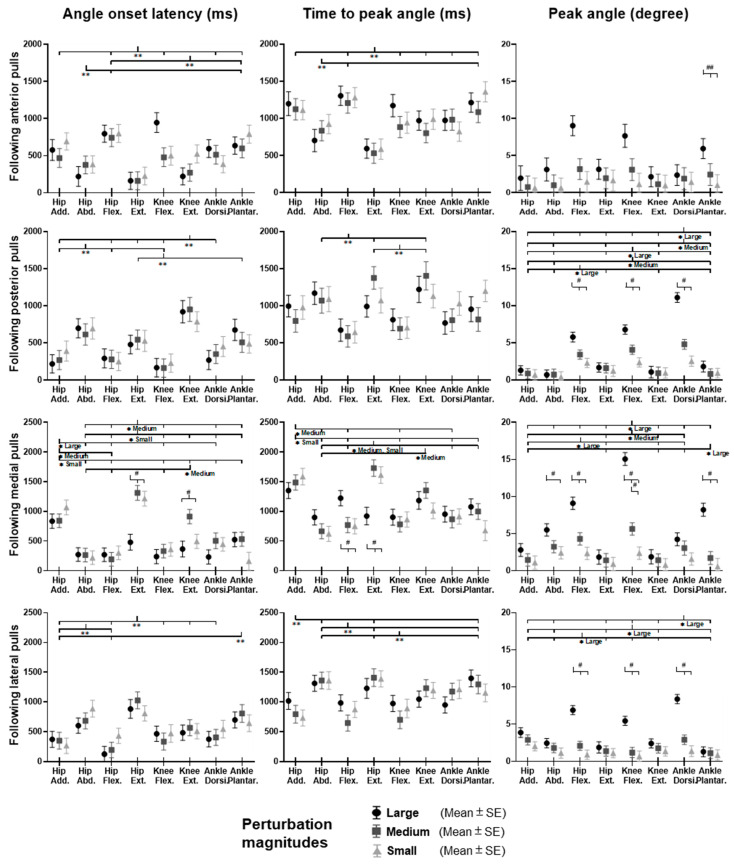
The angle onset latencies, time to peak angle, and peak angles of eight lower-limb joint motions following unexpected horizontal perturbations (mean ± SE, *n* = 12). (Note: **SE**: standard error; 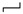 or 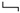: pairwise comparison. Significant differences in post hoc pairwise comparisons (*p* < 0.05) were indicated by the: ** for the main effect of joint motion factor; * for the simple main effect of joint motion factor; **##** for the main effect of magnitude factor; **#** for the simple main effect of magnitude factor).

Figure 5 shows the dominant-leg joint angle changes following the unexpected waist-pull perturbations. As shown in Figure 6, following the unexpected anterior perturbations, the hip extension angle showed significantly shorter onset latency and time to peak angle than the hip flexion angle (*p* < 0.05). Peak angles were not significantly different among the eight joint motions. The larger perturbation magnitudes evoked significantly larger peak angles (*p* < 0.05).

Following the unexpected posterior perturbations, significant within-joint differences were observed in the angle onset latency (knee flexion < extension; hip adduction < abduction; *p* < 0.05) and the time to peak angle (knee flexion < extension; hip flexion < extension; *p* < 0.05). The larger perturbation magnitudes evoked significantly larger peak angles in ankle dorsiflexion, knee flexion, and hip flexion (*p* < 0.05). Under the large magnitude, peak angles of these three joint motions were significantly larger than the other five joint motions (*p* < 0.05). 

Following the unexpected medial perturbations, the hip abduction angle showed significantly shorter onset latency than the hip adduction angle irrespective of perturbation magnitudes (*p* < 0.05). Under the medium and the small magnitudes, significant within-joint differences were observed in the angle onset latency (hip flexion < extension; *p* < 0.05) and the time to peak angle (hip abduction < adduction; hip flexion < extension; *p* < 0.05). Under the medium magnitude, the knee flexion angle showed significantly shorter onset latency than the knee extension angle (*p* < 0.05). The larger perturbation magnitudes evoked significantly larger peak angles in ankle plantarflexion, knee flexion, hip flexion, and hip abduction (*p* < 0.05).

Following the unexpected lateral perturbations, significant within-joint differences were observed in the angle onset latency (hip flexion < extension; hip adduction < abduction; *p* < 0.05) and the time to peak angle (hip flexion < extension; hip adduction < abduction; *p* < 0.05). Under the large magnitude, peak angles of ankle dorsiflexion, knee flexion, and hip flexion were significantly larger than the other five joint motions (*p* < 0.05).

**Figure 7:**
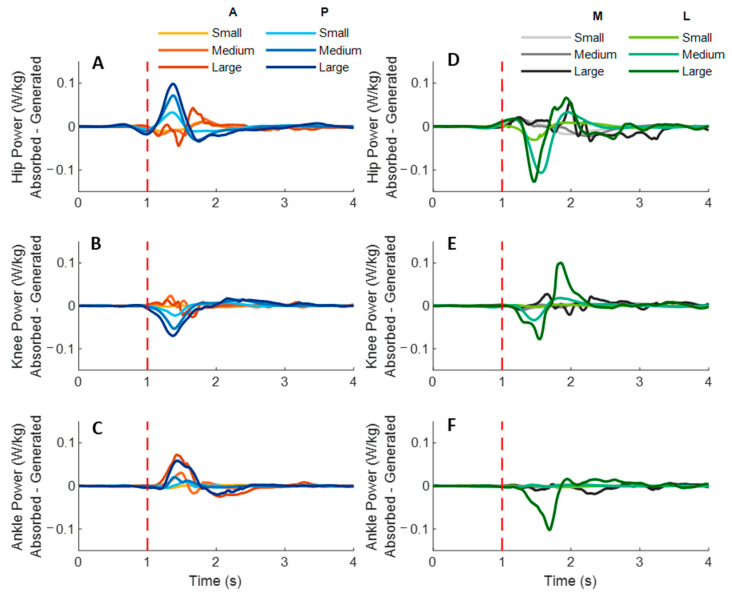
The mean dominant-leg joint power changes of twelve participants following the unexpected anterior, posterior, medial, and lateral perturbations with three magnitudes (*n* = 12). Mean hip (**A**), knee (**B**), and ankle (**C**) power generation and absorption following anterior and posterior perturbations; Mean hip (**D**), knee (**E**), and ankle (**F**) power generation and absorption following medial and lateral perturbations. (Note: The red dotted line indicated the start of pulling perturbation. **A**: anterior; **P**: posterior; **M**: medial; **L**: lateral).

**Figure 8:**
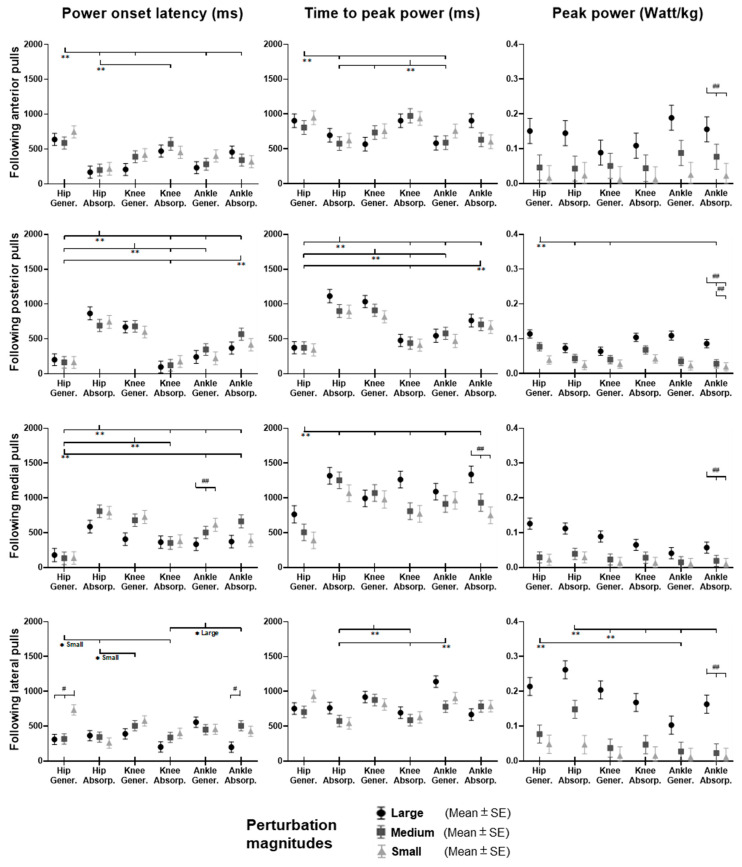
The power onset latencies, time to peak power, and peak power responses in lower-limb joints following unexpected horizontal perturbations (mean ± SE, *n* = 12). (Note: **Gener.**: power generation; **Absorp.**: power absorption; **SE**: standard error; 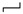 or 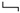: pairwise comparison. Significant differences in post hoc pairwise comparisons (*p* < 0.05) were indicated by the: ** for the main effect of joint motion factor; * for the simple main effect of joint motion factor; **##** for the main effect of magnitude factor; **#** for the simple main effect of magnitude factor).

Figure 7 shows the dominant-leg joint power changes following the unexpected waist-pull perturbations. As shown in Figure 8, following the unexpected anterior perturbations, significant within-joint differences existed in the power onset latency (hip power absorption < generation; *p* < 0.05) and the time to peak power (hip power absorption < generation; knee power generation < absorption; *p* < 0.05). Peak power responses in the hip, knee, and ankle joints were not significantly different. The larger perturbation magnitudes evoked significantly larger peak power responses (*p* < 0.05).

Following the unexpected posterior perturbations, significant within-joint differences existed in the power onset latency (hip power generation < absorption; knee power absorption < generation; *p* < 0.05) and the time to peak power (hip power generation < absorption; knee power absorption < generation; *p* < 0.05). Peak power generated in the hip joint was significantly larger than the absorbed (*p* < 0.05). The larger perturbation magnitudes evoked significantly larger peak power responses (*p* < 0.05). 

Following the unexpected medial perturbations, significant within-joint differences were observed in the power onset latency (hip power generation < absorption; knee power absorption < generation; *p* < 0.05). Hip power generation showed the shortest time to peak among the six lower-limb joint power responses (*p* < 0.05). Peak power responses in the hip, knee, and ankle joints were not significantly different. Generally, the larger perturbation magnitudes evoked significantly shorter power onset latency, longer time to peak power, and larger peak power (*p* < 0.05).

Following the unexpected lateral perturbations, significant within-joint differences in power onset latency were observed under the small (hip power absorption < generation; *p* < 0.05) and the large magnitudes (ankle power absorption < generation; *p* < 0.05). Knee power absorption showed a significantly shorter time to peak than a generation (*p* < 0.05). Peak power absorbed in the hip joint was significantly larger than the peak power responses in knee and ankle joints (*p* < 0.05). The larger perturbation magnitudes evoked significantly larger peak power responses (*p* < 0.05).

### 3.3. Lower-Limb Joint Moments

**Figure 9:**
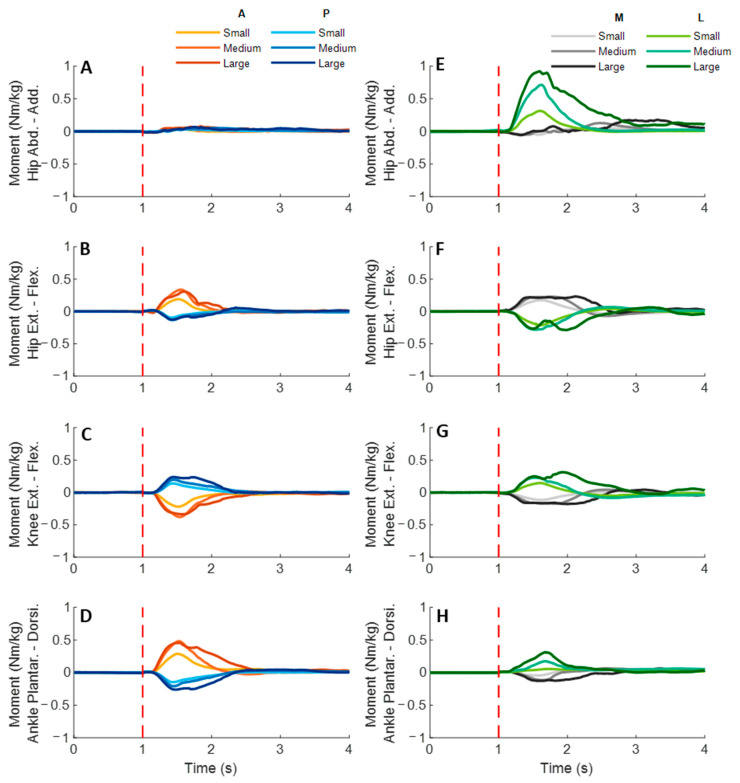
The mean dominant-leg joint moment changes of twelve participants following the unexpected anterior, posterior, medial, and lateral perturbations with three magnitudes (*n* = 12). Mean hip adduction-abduction (**A**), hip flexion-extension (**B**), knee flexion-extension (**C**), and ankle dorsiflexion-plantarflexion (**D**) moment changes following anterior and posterior perturbations; Mean hip adduction-abduction (**E**), hip flexion-extension (**F**), knee flexion-extension (**G**), and ankle dorsiflexion-plantarflexion (**H**) moment changes following medial and lateral perturbations. (Note: The red dotted line indicated the start of pulling perturbation. **Add.**: adduction; **Abd.**: abduction; **Flex.**: flexion; **Ext.**: extension; **Dorsi.**: dorsiflexion; **Plantar.**: plantarflexion. **A**: anterior; **P**: posterior; **M**: medial; **L**: lateral).

**Figure 10:**
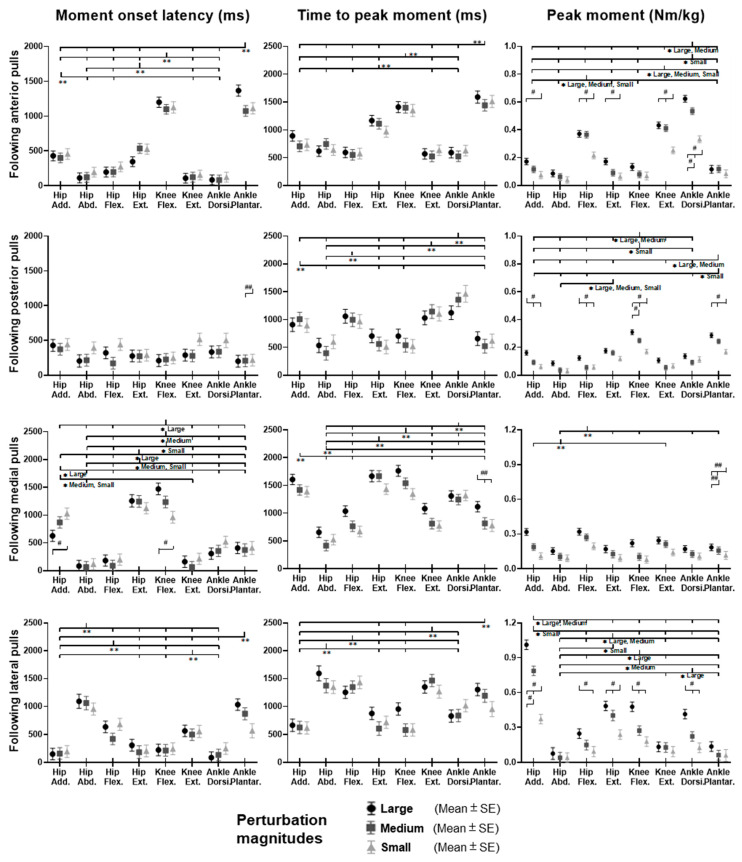
The moment onset latencies, time to peak moment, and peak moments of eight lower-limb joint motions following unexpected horizontal perturbations (mean ± SE, *n* = 12). (Note: **SE**: standard error; 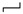 or 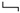: pairwise comparison. Significant differences in post hoc pairwise comparisons (*p* < 0.05) were indicated by the: ** for the main effect of joint motion factor; * for the simple main effect of joint motion factor; **##** for the main effect of magnitude factor; **#** for the simple main effect of magnitude factor).

Figure 9 shows the dominant-leg joint moment changes following the unexpected waist-pull perturbations. As shown in Figure 10, following the unexpected anterior perturbations, significant within-joint differences existed in the moment onset latency (ankle dorsiflexion < plantarflexion; knee extension < flexion; hip flexion < extension; hip adduction < abduction; *p* < 0.05) and the time to peak moment (ankle dorsiflexion < plantarflexion; knee extension < flexion; hip flexion < extension; *p* < 0.05). Peak moments in ankle dorsiflexion, knee extension, and hip flexion were significantly larger than those in the other five joint motions (*p* < 0.05). Particularly, under the medium and large magnitudes, the peak moment of ankle dorsiflexion was the largest among the eight joint motions (*p* < 0.05).

Following the unexpected posterior perturbations, eight joint motions showed no significantly different moment onset latencies. Significant within-joint differences existed in the time to peak moment (ankle plantarflexion < dorsiflexion; knee flexion < extension; hip extension < flexion; hip abduction < adduction; *p* < 0.05). The peak moment of ankle plantarflexion was significantly larger than that of ankle dorsiflexion irrespective of perturbation magnitudes (*p* < 0.05). Knee flexion showed a significantly larger peak moment than knee extension under the medium and the large magnitudes (*p* < 0.05).

Following the unexpected medial perturbations, significant within-joint differences existed in the moment onset latency (hip abduction < adduction; knee extension < flexion; hip flexion < extension; *p* < 0.05). Hip abduction showed the shortest time to peak moment among the eight joint motions (*p* < 0.05). Besides, significant within-joint differences existed in the time to peak moment (hip flexion < extension, knee extension < flexion; ankle plantarflexion < dorsiflexion; *p* < 0.05) and the peak moment (hip flexion > extension; hip adduction > abduction; *p* < 0.05).

**Figure 11:**
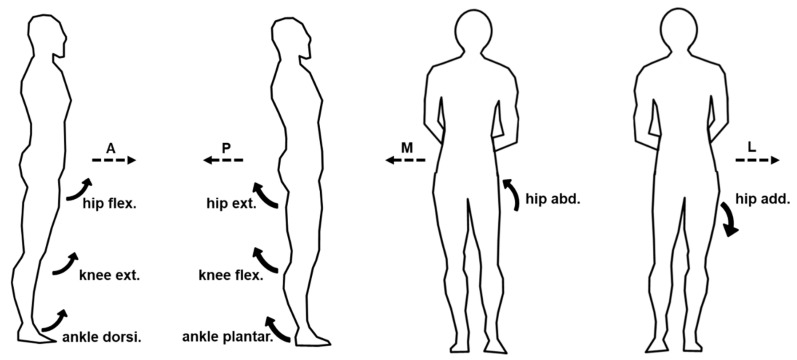
Rapid lower-limb joint moment responses evoked by the unexpected waist-pull perturbations. (Note that the right leg was the dominant leg. **A**: anterior pulls; **P**: posterior pulls; **M**: medial pulls; **L**: lateral pulls).

Following the unexpected lateral perturbations, significant within-joint differences existed in the moment onset latency (hip adduction < abduction; hip extension < flexion; knee flexion < extension; ankle dorsiflexion < plantarflexion; *p* < 0.05) and the time to peak moment (hip adduction < abduction; hip extension < flexion; knee flexion < extension; *p* < 0.05). Under the medium and large magnitudes, the peak moment of hip adduction was the largest among the eight joint motions (*p* < 0.05). In the sagittal plane, significant differences of peak moments were observed under the large (hip extension > flexion; knee flexion > extension; ankle dorsiflexion > plantarflexion; *p* < 0.05) and the medium (hip extension > flexion; *p* < 0.05) magnitudes. Figure 11 summarized in what joint motions the more rapid moment response would occur following the four directions of waist-pull perturbations.

### 3.4. EMG Signals of Eight Lower-Limb Muscles

**Figure 12:**
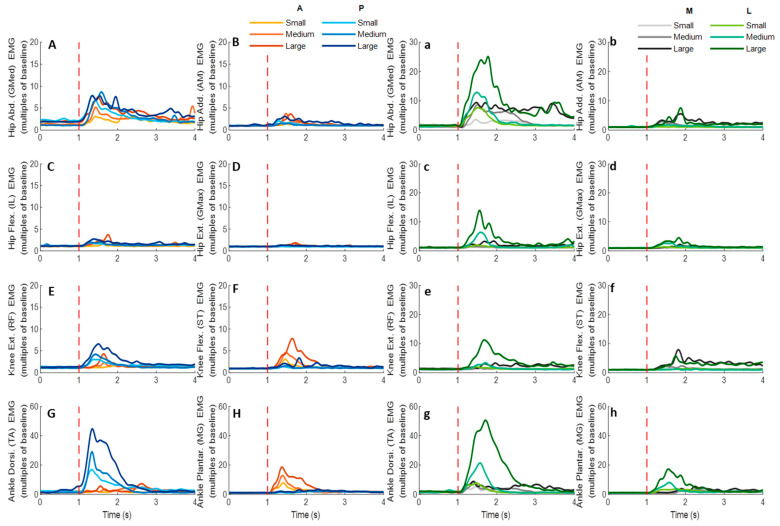
The mean EMG signal changes of twelve participants for eight dominant-leg muscles following the unexpected anterior, posterior, medial, and lateral perturbations with three magnitudes (*n* = 12). Mean EMG signal changes for hip abductor-adductor (**A**,**B**), hip flexor-extensor (**C**,**D**), knee extensor-flexor (**E**,**F**), and ankle dorsiflexor-plantarflexor (**G**,**H**) following anterior and posterior perturbations; Mean EMG signal changes of hip abductor-adductor (**a**,**b**), hip flexor-extensor (**c**,**d**), knee extensor-flexor (**e**,**f**), and ankle dorsiflexor-plantarflexor (**g**,**h**) following medial and lateral perturbations. (Note: The EMG amplitude values were multiples of the 1000-ms baseline mean value before a pulling perturbation. The red dotted line indicated the start of pulling perturbation. **EMG**: electromyography. **GMed**: gluteus medius; **AM**: adductor magus; **IL**: iliopsoas; **GMax**: gluteus maximus; **RF**: rectus femoris; **ST**: semitendinosus; **TA**: tibialis anterior; **MG**: gastrocnemius medialis; **A**: anterior; **P**: posterior; **M**: medial; **L**: lateral).

**Figure 13:**
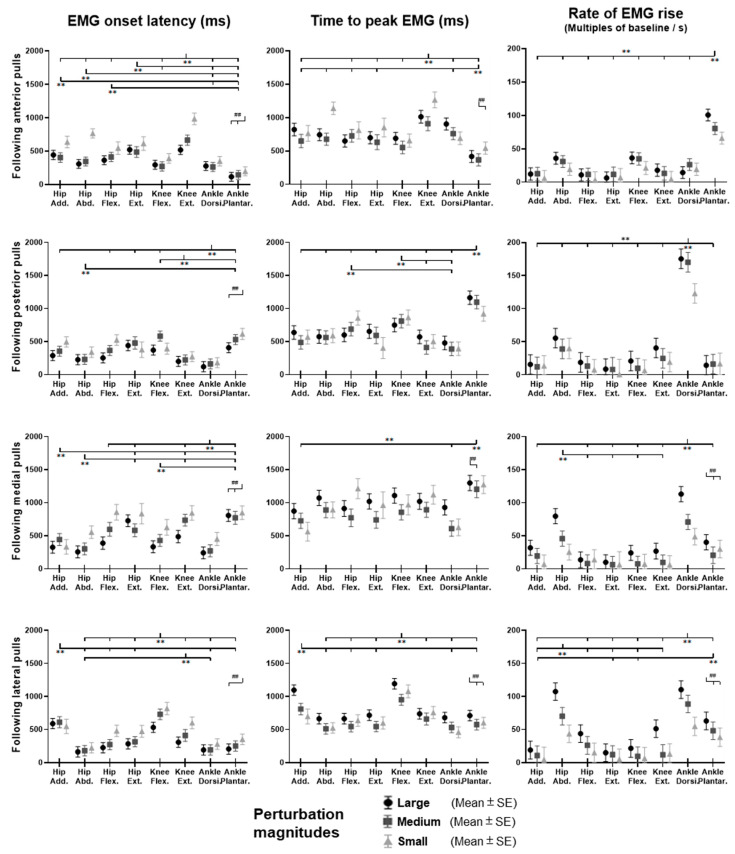
The EMG onset latencies, time to peak EMG amplitude, and rate of EMG rise for eight dominant-leg muscles following unexpected horizontal perturbations (mean ± SE, *n* = 12). (Note: **Hip Add.**: adductor magus; **Hip Abd.**: gluteus medius; **Hip Flex.**: iliopsoas; **Hip Ext.**: gluteus maximus; **Knee Flex.**: semitendinosus; **Knee Ext.**: rectus femoris; **Ankle Dorsi.**: tibialis anterior; **Ankle Plantar.**: gastrocnemius medialis. **SE**: standard error; 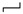 or 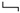: pairwise comparison. Significant differences in post hoc pairwise comparisons (*p* < 0.05) were indicated by the: ** for the main effect of muscle factor; **##** for the main effect of magnitude factor).

Figure 12 demonstrates the dominant-leg muscles’ EMG signal changes following the unexpected waist-pull perturbations. As shown in Figure 13, following the unexpected anterior perturbations, the ankle plantarflexor, ankle dorsiflexor, and knee flexor were in the queue with short EMG onset latencies, and the ankle plantarflexor showed a significantly shorter EMG onset latency than the other five muscles (*p* < 0.05). Significant agonist-antagonist differences existed in the EMG onset latency (knee flexor < extensor; *p* < 0.05) and the time to peak EMG amplitude (ankle plantarflexor < dorsiflexor; knee flexor < extensor; *p* < 0.05). Ankle plantarflexor showed the largest rate of EMG rise among the eight lower-limb muscles (*p* < 0.05). The larger perturbation magnitudes evoked significantly shorter EMG onset latencies and shorter time to peak EMG amplitude (*p* < 0.05).

Following the unexpected posterior perturbations, the ankle dorsiflexor, knee extensor, and hip abductor were in the queue with short EMG onset latencies, and the ankle dorsiflexor showed a significantly shorter EMG onset latency than the other five muscles (*p* < 0.05). Significant agonist-antagonist differences were observed in the EMG onset latency (ankle dorsiflexor < plantarflexor; knee extensor < flexor; *p* < 0.05) and the time to peak EMG amplitude (ankle dorsiflexor < plantarflexor; knee extensor < flexor; *p* < 0.05). The ankle dorsiflexor showed the largest rate of EMG rise among the eight lower-limb muscles (*p* < 0.05). The larger perturbation magnitudes evoked significantly shorter EMG onset latencies (*p* < 0.05).

Following the unexpected medial perturbations ankle dorsiflexor, hip adductor, hip abductor and knee flexor were in the queue with short EMG onset latencies, and the ankle dorsiflexor showed a significantly shorter EMG onset latency than the remaining four muscles (*p* < 0.05). Significant agonist-antagonist difference existed in the EMG onset latency (ankle dorsiflexor < plantarflexor; *p* < 0.05) and the time to peak EMG amplitude (ankle dorsiflexor < plantarflexor; *p* < 0.05). Except for the hip abductor, the ankle dorsiflexor muscle showed a significantly larger rate of EMG rise than the other six muscles (*p* < 0.05). The larger perturbation magnitudes evoked significantly shorter EMG onset latencies, longer time to peak EMG amplitude, and a larger rate of EMG rise (*p* < 0.05).

Following the unexpected lateral perturbations, significant agonist-antagonist differences existed in the EMG onset latency (hip abductor < hip adductor; knee extensor < knee flexor; *p* < 0.05) and the time to peak EMG amplitude (hip abductor < hip adductor; knee extensor < knee flexor; *p* < 0.05). Except for the hip abductor, the ankle dorsiflexor showed a significantly larger rate of EMG rise than the other six muscles (*p* < 0.05). In the frontal plane, the hip abductor showed a significantly larger rate of EMG rise than the hip adductor (*p* < 0.05). The larger perturbation magnitudes evoked significantly shorter EMG onset latencies, longer time to peak EMG amplitude, and a larger rate of EMG rise (*p* < 0.05).

### 3.5. MMG Signals of Eight Lower-Limb Muscles

**Figure 14:**
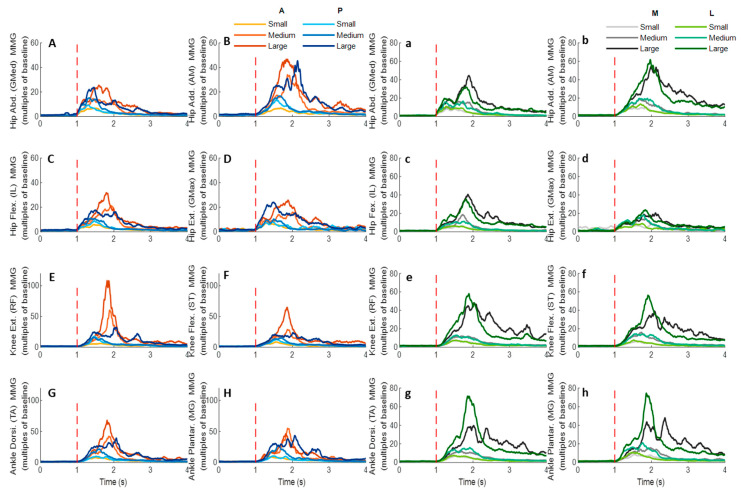
The mean MMG signal changes of twelve participants for eight dominant-leg muscles following the unexpected anterior, posterior, medial, and lateral perturbations with three magnitudes (*n* = 12). Mean MMG signal changes for hip abductor-adductor (**A**,**B**), hip flexor-extensor (**C**,**D**), knee extensor-flexor (**E**,**F**), and ankle dorsiflexor-plantarflexor (**G**,**H**) following anterior and posterior perturbations; Mean MMG signal changes of hip abductor-adductor (**a**,**b**), hip flexor-extensor (**c**,**d**), knee extensor-flexor (**e**,**f**), and ankle dorsiflexor-plantarflexor (**g**,**h**) following medial and lateral perturbations. (Note: The MMG amplitude values were multiples of the 1000-ms baseline mean value before a pulling perturbation. The red dotted line indicated the start of pulling perturbation. **MMG**: mechanomyography. **GMed**: gluteus medius; **AM**: adductor magus; **IL**: iliopsoas; **GMax**: gluteus maximus; **RF**: rectus femoris; **ST**: semitendinosus; **TA**: tibialis anterior; **MG**: gastrocnemius medialis; **A**: anterior; **P**: posterior; **M**: medial; **L**: lateral).

**Figure 15:**
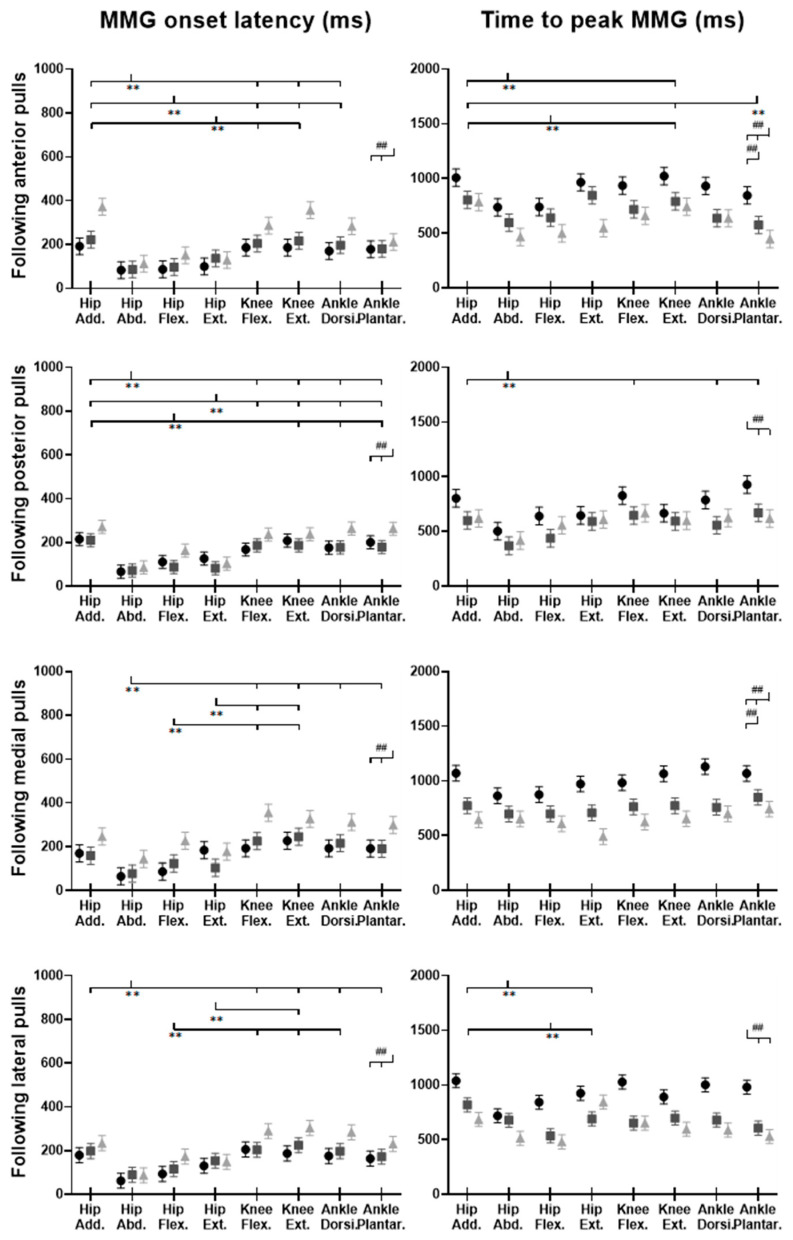
The MMG onset latencies and time to peak MMG amplitude for eight dominant-leg muscles following unexpected horizontal perturbations (mean ± SE, *n* = 12). (Note: **Hip Add.**: adductor magus; **Hip Abd.**: gluteus medius; **Hip Flex.**: iliopsoas; **Hip Ext.**: gluteus maximus; **Knee Flex.**: semitendinosus; **Knee Ext.**: rectus femoris; **Ankle Dorsi.**: tibialis anterior; **Ankle Plantar.**: gastrocnemius medialis. **MMG**: mechanomyography; **SE**: standard error; 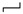 or 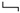: pairwise comparison. Significant differences in post hoc pairwise comparisons (*p* < 0.05) were indicated by the: ** for the main effect of muscle factor; ## for the main effect of magnitude factor).

Figure 14 demonstrates the eight muscles’ MMG signal changes following the unexpected waist-pull perturbations. As shown in Figure 15, following all the four directions of unexpected perturbations, the hip abductor, hip flexor, and hip extensor were in the queue with short MMG onset latencies. Significant agonist-antagonist differences in MMG onset latencies were observed (hip abductor < adductor; *p* < 0.05) following anterior, posterior, and lateral perturbations. The larger perturbation magnitudes evoked significantly shorter MMG onset latencies for all the four pulling directions (*p* < 0.05).

Regarding the time to peak MMG amplitude, significant agonist-antagonist differences were observed following anterior (hip abductor < adductor; *p* < 0.05), posterior (hip abductor < adductor; *p* < 0.05) and lateral (hip abductor < adductor; hip flexor < extensor; *p* < 0.05) perturbations. The larger perturbation magnitudes evoked a significantly longer time to peak MMG amplitude following all the four directions of unexpected perturbations (*p* < 0.05).

## 4. Discussion

With the innovatively synchronized measurement of postural sway, joint kinetics and kinematics, and muscle EMG and MMG activities, this study comprehensively investigated and uncovered how hip, knee, and ankle muscles and joints reacted to the unexpected perturbations in sagittal and frontal planes. Generally, this study observed that: (1) agonist muscles that resisted the perturbation had more rapid activation than the antagonist muscles; (2) among all agonist muscles resisting the perturbation, ankle muscles had the largest rate of activation in the sagittal or frontal plane; (3) CoP and lower-joint moments that followed the perturbation had faster increase; and (4) the larger magnitude of perturbations tended to induce faster responses in muscle activities, but not necessarily in joint motions. These findings not only build on our knowledge of how lower-limb muscles and joints respond to balance perturbations, but also facilitate future applied research on developing the targeted balance exercise program and/or the (robotic) assistive technologies/devices to prevent falls of older people and patients. More details can be found below.

### 4.1. Faster Activation Occurred in Muscles Resisting Perturbations, Especially for Ankle Muscles

The primary finding of this study is that more rapid activation existed in the agonist muscles that resisted the pulling perturbations, as compared to the antagonist muscles; and ankle muscles appeared to have the earliest and most rapid activation in response to the perturbations in either sagittal (anterior & posterior) or frontal (medial & lateral) plane. 

This study observed that for anterior perturbation, muscles moving the body posteriorly (ankle plantarflexor, knee flexor) had early activation and reached the peak neuromuscular activation early. This is consistent with the previous finding that dorsal leg muscles (gastrocnemius, hamstrings) had earlier onset of reflexive activities than ventral muscles, following the unexpected perturbations induced by a backward-moving platform [9]. The ankle plantarflexor also showed the largest rate of neuromuscular activation among the eight muscles in this study. The rate of EMG rise in the early phase (50 ms after the EMG onset) has been reported as one key determinant of rapid force generation [41], and a large rate of dorsal leg muscles’ activation was important for preventing tripping [13]. This study further suggested that among the eight lower-limb muscles, the ankle plantarflexor had the most rapid increase of muscle activities to resist the excessive anterior pulling perturbations. 

Similarly, this study observed that for posterior perturbation, muscles moving the body anteriorly (ankle dorsiflexor, knee extensor) had earlier activation and reached the peak neuromuscular activation earlier than their antagonist muscles. Such results are consistent with the previous studies that found shorter EMG onset latencies [16,17,42] and time to peak EMG amplitude [17] existed in the ventral leg muscles (TA and RF), following the unexpected perturbations induced by a forward-moving platform. Furthermore, this study also observed that the ankle dorsiflexor had a significantly larger rate of neuromuscular activation than the other seven lower-limb muscles. While limited previous studies investigated the rate of neuromuscular activation following balance perturbations, the findings of this study suggested that the ankle dorsiflexor was activated most rapidly in response to the posterior perturbation. 

This study also observed that for medial perturbation, the hip adductor and hip abductor had earlier activation; and for lateral perturbation, more lower-limb muscles, including the hip abductor, had earlier activation since more body weight was transferred to the dominant leg. This supported the previous studies’ finding that the declined rate of hip abductor/adductor activation correlated with a lower incidence of protective stepping following the unexpected lateral waist-pulls [8]. On top of this, this study further found that the ankle dorsiflexor’s rapid neuromuscular activation is essential for maintaining the mediolateral standing balance.

### 4.2. Postural Sway and Joint Moment Response Followed Perturbations

The secondary finding of this study is that the CoP took less time to reach the peak displacement and had a larger peak displacement than the CoM, and the joint moments that resisted the perturbation had an earlier and faster increase following the perturbation.

This agrees with the inverted pendulum assumption that the distance between CoP and CoM displacements was correlated to the CoM acceleration [6,43]. By moving the CoP quickly in the same direction as the sudden CoM displacement, the change in CoM would be decelerated and kept within the BoS [6]. Based on the current findings, it is also anticipated that the onset sequence of CoM and CoP may depend on the pulling direction. The anterior, medial, and lateral perturbation induced earlier onset of CoP, and the posterior perturbation induced earlier onset of CoM. This may be because the posterior perturbation is less anticipated and poses a higher risk of uncertainty/falls for participants, as compared to the other three directions. The previous study also reported earlier CoM displacement following the unexpected standing perturbations, and earlier CoP displacement following the anticipated perturbations [44]. Concerning this, the findings of this study may further suggest that more reaction time is needed for making the compensatory postural adjustment (CPA) following the posterior perturbation or backward loss of balance.

The observation that quicker and larger joint moments occurred to comply with the perturbation direction further supported the above-mentioned postural sway trend (i.e., CoP and CoM displacements). Specifically, for anterior perturbation, the ankle dorsiflexion, knee extension and hip flexion moments showed earlier onset, reached peaks faster, and reached larger peaks. Consequently, these joint moments drove the pelvis, thigh, and shank anteriorly, resulting in anterior CoP displacement. This is contrary to a previous study reporting earlier responses of ankle plantarflexion, knee extension, and hip extension moments following perturbation induced by a backward-moving platform, which caused also the sudden forward CoM displacement with respect to the BoS [9]. This may be due to the different perturbation methods and magnitudes. The waist-pull perturbation in this study exerted perturbation at the proximal body part (at the pelvis), while that of using a moving platform generated perturbation at the distal body part (at the foot). Further studies are needed to compare the two perturbation methods and verify this.

Similarly, this study observed that posterior perturbation induced a quicker response in ankle plantarflexion, knee flexion, and hip extension moments to move lower limbs posteriorly. This finding is comparable to a previous study reporting earlier responses of ankle plantarflexion, knee extension, and hip extension moments, following posterior perturbation induced by a backward-moving platform [9]. The different reaction at knee joint may be explained by the strategy in participants, where they may try to further lower the CoM by flexing the knee joints. These findings provide evidence of the joint moment changes, in response to the posterior standing perturbations and sudden backward CoM displacement, which may have been unclear/unavailable previously.

For medial perturbation, this study observed an earlier increase and earlier reaching of the peak for hip abduction moment, and a larger peak moment for hip adduction. This is consistent with a previous study, which observed sinusoidal response of hip adduction/abduction moment following inward pushes of the pelvis [6]. The firstly appeared increase of hip abduction moment may contribute to the quick medial CoP displacement, while the latter increase of hip adduction moment may be functioned to restore the CoP laterally and back to the dominant leg. The observed earlier/quicker moment increase in hip flexion, knee extension, and ankle plantarflexion of the dominant leg may add more evidence on the joint moment responses of the sagittal plane to the medial perturbations. 

For lateral perturbation, this study observed an earlier, quicker, and larger increase of hip adduction moment, leading to lateral CoP displacement. This echoes the previous study that reported increased corrective hip abduction moment after lateral pushes on the pelvis [6]. Additionally, this study observed the earlier and quicker increase of hip extension, knee flexion, and/or ankle dorsiflexion moments in the sagittal plane. While the faster response of knee flexion moment occurred in both the posterior and the lateral perturbation directions in this study, future studies are needed to verify if the knee flexion moment has functioned to lower the CoM and maintain standing balance by investigating the superior-inferior or vertical movement of CoM following a perturbation. These findings build on our knowledge and understanding regarding the detailed CoP, CoM, and joint moment reactions immediately after the perturbations.

### 4.3. Lower-Limb Responses Tended to Be Affected by the Varying Perturbation Magnitudes

The tertiary finding of this study is that in general, the rapid responses of lower-limb muscle activities tended to be proportional to the perturbation magnitude levels. More specifically, this study observed that the larger magnitude of perturbations evoked earlier onset of lower-limb muscle EMG and MMG activities, following all four directions of waist-pull perturbations. This was consistent with the previous finding that the increasing magnitude of forward [16,45] and backward [9] moving-platform perturbation could result in shorter EMG onset latencies of leg muscles, but was contrary to another study that found no effects of varying perturbation magnitudes on the leg muscles’ EMG onset latencies (anterior & posterior) [46]. The disparity may be caused by the different range of velocities used for perturbation magnitudes. On top of the previous findings, this study supported that in the frontal plane (medial & lateral), earlier onset of muscle activities may also be evoked by the larger perturbation magnitude. Further, this study observed that the larger magnitude of perturbations evoked a larger rate of EMG activation, and a longer time to peak muscle EMG and MMG activities, following medial and lateral perturbations. To the knowledge of the authors, previous studies reported little on the rate of EMG rise and the time to peak muscle activity in response to the different levels of balance perturbations. These results collectively suggested that for young adults, the lower-limb muscle activities appeared to have the below responses to accommodate a larger magnitude of waist-pull balance perturbation: starting earlier, increasing faster immediately after start, and keeping in activation for a longer time.

Similar to the previous findings [9,15,46], the peak responses of CoM displacement, CoP displacement, lower-limb joint moments, power, and angles were observed to be proportional to the perturbation magnitudes in this study. By contrast, this trend was not observed for the rapid responses of these parameters which appeared to vary for different perturbation directions. The perturbation magnitudes were position- and velocity-controlled in this study, and the pulling durations of “small”, “medium” and “large” magnitudes were set to be the same. This may explain why the onset latencies and time to peak following some directions of pulls were not proportional to the perturbation magnitudes. Nevertheless, following the medial perturbation, lower-limb joint rapid responses were all found to be affected by the different perturbation magnitudes. This may account for why the stepping strategies and the foot elevations were more frequently observed under the large magnitude of the medial perturbation. Future studies can be conducted to verify this. 

In addition, this study could be innovative in using the balance perturbations that were tailored to the participant’s stature. Some previous studies have attempted to normalize the force of perturbation to the bodyweight [7]. However, regarding the position-controlled perturbations, very few attempts have been made to minimize the possible confounding effects of body height. The different perturbation magnitudes, i.e., pulling displacements, were divided by the participant’s height in this study, which may make the finding of different perturbation magnitudes’ effects on balance response more reliable and generalizable.

### 4.4. Rapid Power and Angle Responses Were Consistent in Proximal Joints

Regarding the joint angle and power responses, this study observed that the unexpected waist-pull perturbations would evoke the rapid power and angle responses more consistently in hip and/or knee joints, which are proximal lower-limb joints. Joint power was calculated by multiplying the angular velocity with the joint moment. Thus, the power generation would indicate a joint’s accelerating motion, and the power absorption would indicate a joint’s decelerating motion. The onset latency and time to peak results of this study may thus suggest that the anterior, posterior, medial, and lateral perturbations would evoke an earlier hip decelerating extension motion, earlier knee decelerating flexion motion, earlier hip accelerating abduction motion, and earlier abduction and flexion motions, respectively. Previous studies have rarely reported the onset sequence or the sequence of reaching a peak in hip, knee, and ankle joint motions following waist-pull perturbations. One study reported that the suddenly forward-moving platform evoked early joint motions of ankle plantarflexion, knee extension, and hip extension [16], which has been different from the early onset of joint motions following anterior/posterior waist-pulls in this study. Such differences may be caused by the different perturbation locations. Consistent rapid response of joint angle and power at the proximal lower-limb joints may be because the pulling perturbations were exerted on the pelvis. The proprioceptive receptors in hip and/or knee joints may detect the perturbation signal earlier, leading to more consistent compensatory responses than the ankle joint. Further studies are needed to verify this.

### 4.5. Rapid Response of MMG Signals Occurred in Hip Muscles

This study applied the MMG technology, in an attempt to preliminarily investigate the muscle mechanical activities in response to the sudden perturbations. The detected MMG onset latencies were earlier than those of EMG signals, which did not adhere to the temporal sequence that the onset of electrical activity measured by EMG should precede the onset of muscle vibration measured by MMG [21,40]. This indicates that the detected rapid response of MMG signals in this study may not be generated by the active and voluntary muscle contraction, but by the passive and involuntary muscle movement following the waist-pull perturbation instead. This is further supported by the observed earliest MMG onset latencies at hip muscles, which have been the closest to the perturbation location in this study. While previous studies have reported the reliable use of MMG to reflect the onset of muscle’s voluntary isometric or concentric contractions in sitting and static positions [40,47], this study preliminarily applied it in standing and dynamic situations. However, it should be noted that the current processing method of MMG signals was not able to exclude the noise of passive body-segment movements caused by waist-pull perturbations, and the presented results were not generated by the active and voluntary muscle contraction in response to the sudden waist-pull perturbation. Previous studies have also reported that the location of the sensor influenced the captured MMG signals [48]. Further optimization of the algorithm and experimental set-up is needed to identify an optimal MMG sensor location and achieve the accurate estimation of lower-limb muscles’ active and voluntary rapid contractile responses during dynamic standing situations in the future. The findings of this study on MMG data may serve as a steppingstone and inspire future studies. It may also help to apply some ultrafast imaging technologies to visualize the muscle activity from outside to inside of the human body [40,49,50,51,52,53,54].

### 4.6. Limitations

There are several limitations of this study. Firstly, this study normalized the EMG or MMG signals with reference to the baseline value during unperturbed standing. After carefully reviewing the Consensus for Experimental Design in Electromyography (CEDE) recommendations [55] and the current study’s protocol, the current practice of amplitude normalization may be acceptable. However, considering the leg muscles’ rapid activation, e.g., rate of EMG rise, would be affected by the normalization method, future efforts should be made to identify an optimal normalization procedure of the EMG/MMG signals in balance-perturbation-related studies. 

Secondly, the EMG sensor placement in this study was based on clinical practice and somewhat obsolete. Future studies shall optimize the EMG electrode locations based on the innervation zone of each muscle [56]. It is also possible that the crosstalk between the EMG of the investigated muscles may exist in this study, although such crosstalk shall be minimal, since the anatomical positions of investigated muscles, the locations of EMG sensor placement, and the design of EMG sensors have been carefully reviewed and determined based on the available guidelines in this study. 

Thirdly, it appeared that the processed onset latencies of MMG signals in this study were due to the inertia and involuntary muscle movement following the sudden waist-pulling passively, rather than the active and voluntary muscle contraction in response to the perturbation. More efforts are needed to look into how to distinguish and extract the MMG signals generated from the active and voluntary muscle contraction from those generated from the passive and involuntary muscle movement in the future. 

Another limitation is that a small number of healthy young participants were recruited in this pilot study. It should be noted that the range of 12 participants’ lower-limb responses was generally large for the captured signals, except for the postural sway signals. The large range could partly be caused by the sampling error of small sample size. A larger sample size will be needed to reduce the effects of between-individual difference on the outcomes. In addition, the specific sudden pulling direction and magnitude was randomized and blinded to each participant during the experiment, and the mean value of the three repeated perturbation trials was used for statistical analysis in this study. It is so far unclear how the first trial reaction may influence the results and may be investigated in the future.

## 5. Conclusions

This study observed that the agonist muscles resisting perturbation had more rapid activation than the antagonist muscles; among all agonist muscles resisting the perturbation, the ankle muscles had the largest rate of activation in the sagittal or frontal plane; the postural sway and joint moments that followed the perturbation had earlier and faster increase; and larger magnitude of perturbations tend to induce earlier responses in muscle activities, but not necessarily in joint motions in healthy young adults. These findings enriched our knowledge of how multiple lower-limb muscles and joints coordinated to quickly make compensatory postural adjustments (CPAs), and highlighted the important role of ankle muscles’ rapid response in maintaining reactive standing balance. 

## Figures and Tables

**Table 1:** Investigated muscles and locations of EMG sensor placement.

Muscle	Location
**Ankle dorsiflexor:** tibialis anterior (TA)	at 1/3 on the line between the tip of the fibula and the tip of the medial malleolus.
**Ankle plantarflexor:** medial gastrocnemius (MG)	on the most prominent bulge of the muscle.
**Knee extensor:** rectus femoris (RF)	halfway between the anterior superior iliac spine (ASIS) and the superior boarder of the patella.
**Knee flexor:** semitendinosus (ST)	halfway between the ischial tuberosity and the medial epicondyle of the tibia.
**Hip flexor:** iliopsoas (IL)	at 3–5 cm distal from the ASIS [27].
**Hip extensor:** gluteus maximus (GMax)	halfway between the sacral vertebrae and the greater trochanter.
**Hip abductor:** gluteus medius (GMed)	halfway between the iliac crest to the greater trochanter.
**Hip adductor:** adductor maximus (AM)	halfway between the pubic tubercle and the medial femoral epicondyle [28].

**Table 2:** Demographic data (mean ± SD) of twelve participants.

	Female (*n* = 6)	Male (*n* = 6)	Total (*n* = 12)
Age (year)	21.0 ± 0.6	20.8 ± 0.8	20.9 ± 0.7
Height (cm)	165.3 ± 4.8	174.5 ± 5.6	169.9 ± 6.9
Body mass (kg)	57.3 ± 2.6	59.3 ± 8.7	58.3 ± 6.2
BMI (kg/m^2^)	21.0 ± 1.2	19.4 ± 2.0	20.2 ± 1.8
Dominant leg	Right (*n* = 6)	Right (*n* = 6)	Right (*n* = 12)
Leg length (cm)	85.2 ± 3.2	88.8 ± 4.6	87.0 ± 4.3
IPAQ-S (Kcal/week) ^1^	2089.4 ± 1965.5	2451.5 ± 1994.6	2270.5 ± 1897.4
FES-I short version ^2^	10.2 ± 1.2	11.2 ± 4.8	10.7 ± 3.3

^1^ International Physical Activity Scale—Short version. ^2^ Fall Efficacy Scale—International.

**Table 3:** Mean and ICC values of pulling parameters examining the reliability of the waist-pull system (*n* = 12).

		Duration (s)		Max. Displacement (cm)		Normalized Max. Displacement (%Height)		Max. Velocity (m/s)	
**Direction**	**Magnitude**	**Mean**	**ICC**	**Mean**	**ICC**	**Mean**	**ICC**	**Mean**	**ICC**
Anterior	Large	0.396	0.978 *	11.4	0.987 *	6.7%	0.971 *	0.349	0.908 *
	Medium	0.373	0.993 *	7.4	0.991 *	4.4%	0.983 *	0.238	0.995 *
	Small	0.347	0.984 *	3.8	0.962 *	2.2%	0.927 *	0.127	0.778 *
Posterior	Large	0.263	0.981 *	7.0	0.927 *	4.1%	0.847 *	0.338	0.829 *
	Medium	0.247	0.978 *	4.7	0.964 *	2.8%	0.909 *	0.239	0.857 *
	Small	0.231	0.968 *	2.4	0.983 *	1.4%	0.956 *	0.124	0.994 *
Medial	Large	0.531	0.984 *	16.0	0.950 *	9.5%	0.681 *	0.351	0.716 *
	Medium	0.498	0.993 *	10.5	0.956 *	6.2%	0.730 *	0.252	0.759 *
	Small	0.465	0.984 *	5.3	0.964 *	3.1%	0.807 *	0.136	0.902 *
Lateral	Large	0.530	0.954 *	15.1	0.998 *	8.9%	0.863 *	0.317	0.982 *
	Medium	0.498	0.967 *	9.9	0.967 *	5.8%	0.931 *	0.240	0.943 *
	Small	0.465	0.914 *	4.9	0.995 *	2.9%	0.869 *	0.128	0.715 *

ICC: intraclass correlation coefficient. * Significant difference existed in the intraclass correlation coefficient test (*p* < 0.05).

## Data Availability

Data is contained within the article and supplementary material.

## Supplementary Materials

The following supporting information can be downloaded at: www.mdpi.com/article/10.3390/bios12060430/s1, Table S1: The range, mean and standard deviation of outcome values across 12 participants.

## Abbreviations

AM	adductor maximus
ANOVA	analysis of variance
APA	anticipatory postural adjustments
ASIS	anterior superior iliac spine
BBS	Berg Balance Scale
CEDE	Consensus for Experimental Design in Electromyography
CoM	center of body mass
CoP	center of pressure
CPA	compensatory postural adjustment
EMG	electromyography; electromyographical
FES-I	Fall Efficacy Scale-International
GMax	gluteus maximus
GMed	gluteus medius
ICC	intraclass correlation coefficients
IL	iliopsoas
IPAQ-S	International Physical Activity Questionnaire-Short version
MG	medial gastrocnemius
Mini-BEST	Mini Balance Evaluation Systems Test
MMG	mechanomyography; mechanomyographical
POMA	Performance-Oriented Mobility Assessment
PSIS	posterior superior iliac spine
RF	rectus femoris
SD	standard deviation
SD	standard deviation
SPPB	Short Physical Performance Battery
ST	semitendinosus
TA	tibialis anterior

## References

[1] Kalache A., Fu D., Yoshida S., Al-Faisal W., Beattie L., Chodzko-Zajko W., Fu H., James K., Kalula S., Krishnaswamy B. (2007). World Health Organisation Global Report on Falls Prevention in Older Age.

[2] Pai Y.C., Maki B.E., Iqbal K., McIlroy W.E., Perry S.D. (2000). Thresholds for step initiation induced by support-surface translation: A dynamic center-of-mass model provides much better prediction than a static model. J. Biomech..

[3] Chen B., Lee Y.-J., Aruin A.S. (2015). Anticipatory and compensatory postural adjustments in conditions of body asymmetry induced by holding an object. Exp. Brain Res..

[4] Franchignoni F., Horak F., Godi M., Nardone A., Giordano A. (2010). Using psychometric techniques to improve the Balance Evaluation Systems Test: The mini-BESTest. J. Rehabil. Med..

[5] Kanekar N., Aruin A.S. (2014). Aging and balance control in response to external perturbations: Role of anticipatory and compensatory postural mechanisms. Age.

[6] Rietdyk S., Patla A.E., Winter D.A., Ishac M.G., Little C.E. (1999). Balance recovery from medio-lateral perturbations of the upper body during standing. J. Biomech..

[7] Vlutters M., van Asseldonk E.H.F., van der Kooij H. (2018). Lower extremity joint-level responses to pelvis perturbation during human walking. Sci. Rep..

[8] Inacio M., Creath R., Rogers M.W. (2019). Effects of aging on hip abductor-adductor neuromuscular and mechanical performance during the weight transfer phase of lateral protective stepping. J. Biomech..

[9] Runge C.F., Shupert C.L., Horak F.B., Zajac F.E. (1999). Ankle and hip postural strategies defined by joint torques. Gait Posture.

[10] Ma C.Z.-H., Lee W.C.-C. (2017). A wearable vibrotactile biofeedback system improves balance control of healthy young adults following perturbations from quiet stance. Hum. Mov. Sci..

[11] Hsiao-Wecksler E.T. (2008). Biomechanical and age-related differences in balance recovery using the tether-release method. J. Electromyogr. Kinesiol..

[12] Qu X., Hu X., Lew F.L. (2012). Differences in lower extremity muscular responses between successful and failed balance recovery after slips. Int. J. Ind. Ergon..

[13] Pijnappels M., Bobbert M.F., van Dieën J.H. (2005). Control of support limb muscles in recovery after tripping in young and older subjects. Exp. Brain Res..

[14] Pijnappels M., Bobbert M.F., van Dieën J.H. (2005). Push-off reactions in recovery after tripping discriminate young subjects, older non-fallers and older fallers. Gait Posture.

[15] Zemkova E., Kovacikova Z., Jelen M., Neumannova K., Janura M. (2016). Postural and Trunk Responses to Unexpected Perturbations Depend on the Velocity and Direction of Platform Motion. Phys. Res..

[16] Hwang S., Tae K., Sohn R., Kim J., Son J., Kim Y. (2009). The Balance Recovery Mechanisms Against Unexpected Forward Perturbation. Ann. Biomed. Eng..

[17] de Freitas P.B., Knight C.A., Barela J.A. (2010). Postural reactions following forward platform perturbation in young, middle-age, and old adults. J. Electromyogr. Kinesiol..

[18] Blomqvist S., Wester A., Rehn B. (2014). Postural muscle responses and adaptations to backward platform perturbations in young people with and without intellectual disability. Gait Posture.

[19] Bates A.V., McGregor A., Alexander C.M. (2021). Adaptation of balance reactions following forward perturbations in people with joint hypermobility syndrome. BMC Musculoskelet. Disord..

[20] Orizio C. (1993). Muscle sound: Bases for the introduction of a mechanomyographic signal in muscle studies. Crit. Rev. Biomed. Eng..

[21] Cè E., Longo S., Limonta E., Coratella G., Rampichini S., Esposito F. (2020). Peripheral fatigue: New mechanistic insights from recent technologies. Eur. J. Appl. Physiol..

[22] Manchester D., Woollacott M., Zederbauer-Hylton N., Marin O. (1989). Visual, Vestibular and Somatosensory Contributions to Balance Control in the Older Adult. J. Gerontol..

[23] Horak F.B., Nashner L.M. (1986). Central programming of postural movements: Adaptation to altered support-surface configurations. J. Neurophysiol..

[24] Shumway-Cook A., Woollacott M.H. (1995). Motor Control: Theory and Practical Applications.

[25] Vicon Motion Systems; UK. Full Body Modeling with Plug-in Gait. https://docs.vicon.com/display/Nexus213/Full+body+modeling+with+Plug-in+Gait.

[26] Hermens H., Freriks B., Merletti R., Stegeman D.F., Blok J.H., Rau G., Klug C.D., Hägg G., Blok W.J., Hermens H.J. (1999). European recommendations for surface electromyography: Results of the SENIAM Project. Roessingh Res. Dev..

[27] Jiroumaru T., Kurihara T., Isaka T. (2014). Establishment of a recording method for surface electromyography in the iliopsoas muscle. J. Electromyogr. Kinesiol..

[28] Hides J.A., Beall P., Franettovich Smith M.M., Stanton W., Miokovic T., Richardson C. (2016). Activation of the hip adductor muscles varies during a simulated weight-bearing task. Phys. Ther. Sport.

[29] Lee P.H., Macfarlane D.J., Lam T.H., Stewart S.M. (2011). Validity of the International Physical Activity Questionnaire Short Form (IPAQ-SF): A systematic review. Int. J. Behav. Nutr. Phys. Act..

[30] Kempen G.I.J.M., Yardley L., Van Haastregt J.C.M., Zijlstra G.A.R., Beyer N., Hauer K., Todd C. (2008). The Short FES-I: A shortened version of the falls efficacy scale-international to assess fear of falling. Age Ageing.

[31] Hoffman M., Schrader J., Applegate T., Koceja D. (1998). Unilateral postural control of the functionally dominant and nondominant extremities of healthy subjects. J. Athl. Train..

[32] Bair W.-N., Prettyman M.G., Beamer B.A., Rogers M.W. (2016). Kinematic and behavioral analyses of protective stepping strategies and risk for falls among community living older adults. Clin. Biomech..

[33] Pai Y.-C., Rogers M.W., Patton J., Cain T.D., Hanke T.A. (1998). Static versus dynamic predictions of protective stepping following waist–pull perturbations in young and older adults. J. Biomech..

[34] Luchies C.W., Alexander N.B., Schultz A.B., Ashton-Miller J. (1994). Stepping responses of young and old adults to postural disturbances: Kinematics. J. Am. Geriatr. Soc..

[35] Singh H., Sanders O., McCombe Waller S., Bair W.-N., Beamer B., Creath R.A., Rogers M.W. (2017). Relationship Between Head-Turn Gait Speed and Lateral Balance Function in Community-Dwelling Older Adults. Arch. Phys. Med. Rehabil..

[36] Kadaba M.P., Ramakrishnan H.K., Wootten M.E., Gainey J., Gorton G., Cochran G.V.B. (1989). Repeatability of kinematic, kinetic, and electromyographic data in normal adult gait. J. Ortho. Res..

[37] Laudani L., Rum L., Valle M.S., Macaluso A., Vannozzi G., Casabona A. (2021). Age differences in anticipatory and executory mechanisms of gait initiation following unexpected balance perturbations. Eur. J. Appl. Physiol..

[38] Kim D., Hwang J.-M. (2018). The center of pressure and ankle muscle co-contraction in response to anterior-posterior perturbations. PLoS ONE.

[39] Plewa K., Samadani A., Orlandi S., Chau T. (2018). A novel approach to automatically quantify the level of coincident activity between EMG and MMG signals. J. Electromyogr. Kinesiol..

[40] Ling Y.T., Ma C.Z., Shea Q.T., Zheng Y.-P. (2020). Sonomechanomyography (SMMG): Mapping of Skeletal Muscle Motion Onset during Contraction Using Ultrafast Ultrasound Imaging and Multiple Motion Sensors. Sensors.

[41] Folland J.P., Buckthorpe M.W., Hannah R. (2014). Human capacity for explosive force production: Neural and contractile determinants. Scand. J. Med. Sci. Sports.

[42] Tsai Y.-C., Hsieh L.-F., Yang S. (2013). Age-related changes in posture response under a continuous and unexpected perturbation. J. Biomech..

[43] Winter D.A., Patla A.E., Prince F., Ishac M., Gielo-Perczak K. (1998). Stiffness Control of Balance in Quiet Standing. J. Neurophysiol..

[44] Santos M.J., Kanekar N., Aruin A.S. (2010). The role of anticipatory postural adjustments in compensatory control of posture: 2. Biomechanical analysis. J. Electromyogr. Kinesiol. Off. J. Int. Soc. Electrophys. Kinesiol..

[45] Lin S.I., Woollacott M.H. (2002). Postural muscle responses following changing balance threats in young, stable older, and unstable older adults. J. Motor. Behav..

[46] Szturm T., Fallang B. (1998). Effects of varying acceleration of platform translation and toes-up rotations on the pattern and magnitude of balance reactions in humans. J. Vestib. Res..

[47] Smith C.M., Housh T.J., Hill E.C., Johnson G.O., Schmidt R.J. (2017). Dynamic versus isometric electromechanical delay in non-fatigued and fatigued muscle: A combined electromyographic, mechanomyographic, and force approach. J. Electromyogr. Kinesiol..

[48] Cescon C., Farina D., Gobbo M., Merletti R., Orizio C. (2004). Effect of accelerometer location on mechanomyogram variables during voluntary, constant-force contractions in three human muscles. Med. Biol. Eng. Comput..

[49] Ma C.Z.-H., Ling Y.T., Shea Q.T.K., Wang L.-K., Wang X.-Y., Zheng Y.-P. (2019). Towards Wearable Comprehensive Capture and Analysis of Skeletal Muscle Activity during Human Locomotion. Sensors.

[50] Ma C.Z.-H., Ren L.-J., Cheng C.L.-K., Zheng Y.-P. (2020). Mapping of Back Muscle Stiffness along Spine during Standing and Lying in Young Adults: A Pilot Study on Spinal Stiffness Quantification with Ultrasound Imaging. Sensors.

[51] Ren L.J., Wang L.K., Ma C.Z.-H., Yang Y.X., Zheng Y.P. (2019). Effect of Conventional Physiotherapy on Pain and Muscle Stiffness inPatients with Low Back Pain Assessed by a Wireless Hand-held Tissue Ultrasound Palpation System (TUPS). Int. J. Phys. Med. Rehabil..

[52] Ren L.-J., Cheng C.L.-K., Ma C.Z.-H., Zheng Y.-P. (2021). Changes in Muscle Hardness from Resting to Mid-Range Lengthened Positions Detected by Shear Wave Elastography (SWE) with a Novel Protocol of Ultrasound Probe Placement. Appl. Sci..

[53] Huang Z.-H., Ma C.Z.-H., Wang L.-K., Wang X.-Y., Fu S.-N., Zheng Y.-P. (2022). Real-Time Visual Biofeedback via Wearable Ultrasound Imaging Can Enhance the Muscle Contraction Training Outcome of Young Adults. J. Strength Cond. Res..

[54] Lyu P.-Z., Zhu R.T.-L., Ling Y.T., Wang L.-K., Zheng Y.-P., Ma C.Z.-H. (2022). How Paretic and Non-Paretic Ankle Muscles Contract during Walking in Stroke Survivors: New Insight Using Novel Wearable Ultrasound Imaging and Sensing Technology. Biosensors.

[55] Besomi M., Hodges P.W., Clancy E.A., Van Dieën J., Hug F., Lowery M., Merletti R., Søgaard K., Wrigley T., Besier T. (2020). Consensus for experimental design in electromyography (CEDE) project: Amplitude normalization matrix. J. Electromyogr. Kinesiol..

[56] Barbero M., Merletti R., Rainoldi A. (2012). Atlas of Muscle Innervation Zones: Understanding Surface Electromyography and Its Applications.

